# Characterization of Antibiotic Resistance in Select Tertiary Hospitals in Uganda: An Evaluation of 2020 to 2023 Routine Surveillance Data

**DOI:** 10.3390/tropicalmed9040077

**Published:** 2024-04-05

**Authors:** Jonathan Mayito, Daniel Kibombo, Charles Olaro, Susan Nabadda, Consolata Guma, Immaculate Nabukenya, Andrew Busuge, Flavia Dhikusooka, Alex Andema, Peter Mukobi, Nathan Onyachi, Ben Watmon, Stephen Obbo, Alfred Yayi, James Elima, Celestine Barigye, Filbert J. Nyeko, Ibrahim Mugerwa, Musa Sekamatte, Joel Bazira, Richard Walwema, Mohammed Lamorde, Francis Kakooza, Henry Kajumbula

**Affiliations:** 1 Infectious Diseases Institute, Makerere University College of Health Sciences, Kampala P.O. Box 22418, Uganda; dkibombo@idi.co.ug (D.K.); abusuge@gmail.com (A.B.);; 2 Ministry of Health, Kampala P.O. Box 7272, Uganda; 3 Regional Referral Hospital, Ministry of Health, Kampala P.O. Box 7272, Uganda; andemax2002@yahoo.com (A.A.); mukpet@gmail.com (P.M.);; 4 Department of Microbiology, Mbarara University of Science and Technology, Mbarara P.O. Box 1410, Uganda; 5 Department of Microbiology, Makerere University College of Health Sciences, Kampala P.O. Box 7072, Uganda; henrykajumbula427@gmail.com

**Keywords:** antimicrobial resistance, AMR, antibiotic resistance, bacterial antimicrobial resistance, AMR surveillance

## Abstract

Antimicrobial resistance (AMR) is a public health concern in Uganda. We sought to conduct an extended profiling of AMR burden at selected Ugandan tertiary hospitals. We analyzed routine surveillance data collected between October 2020 and March 2023 from 10 tertiary hospitals. The analysis was stratified according to the hospital unit, age, gender, specimen type, and time. Up to 2754 isolates were recovered, primarily from pus: 1443 (52.4%); urine: 1035 (37.6%); and blood: 245 (8.9%). Most pathogens were *Staphylococcus aureus*, 1020 (37%), *Escherichia coli*, 808 (29.3%), and *Klebsiella* spp., 200 (7.3%). Only 28% of *Escherichia coli* and 42% of the other Enterobacterales were susceptible to ceftriaxone, while only 44% of *Staphylococcus aureus* were susceptible to methicillin (56% were MRSA). *Enterococcus* spp. susceptibility to vancomycin was 72%. The 5–24-year-old had 8% lower ampicillin susceptibility than the >65-year-old, while the 25–44-year-old had 8% lower ciprofloxacin susceptibility than the >65-year-old. The 0–4-year-old had 8% higher ciprofloxacin susceptibility. Only erythromycin susceptibility varied by sex, being higher in males. *Escherichia coli* ciprofloxacin susceptibility in blood (57%) was higher than in urine (39%) or pus (28%), as was ceftriaxone susceptibility in blood (44%) versus urine (34%) or pus (14%). *Klebsiella* spp. susceptibility to ciprofloxacin and meropenem decreased by 55% and 47%, respectively, during the evaluation period. During the same period, *Escherichia coli* ciprofloxacin susceptibility decreased by 40%, while *Staphylococcus aureus* gentamicin susceptibility decreased by 37%. Resistance was high across the Access and Watch antibiotic categories, varying with time, age, sex, specimen type, and hospital unit. Effective antimicrobial stewardship targeted at the critical AMR drivers is urgently needed.

## 1. Background

Antimicrobial resistance (AMR) is projected to cause 10 million deaths annually by 2050, ranking it among the leading causes of death and a global health threat [1]. A review of the AMR global burden in 2019 revealed a concerning rise in AMR, with an estimated 1.27 million deaths directly linked to it [2]. This was higher than the mortality due to the Human Immunodeficiency Virus (HIV) or malaria [3], which were the previously leading infectious killers [2]. The AMR burden fell disproportionately on sub-Saharan Africa and Asia, with Western sub-Saharan Africa experiencing the highest all-age AMR-attributable death rate at 27.3 per 100,000. [2]. This coincides with the poor preparedness of the AMR surveillance systems and the paucity of AMR data in these regions [3].

Before 2018, Uganda’s AMR data were from isolated studies, which pointed to an emerging AMR epidemic but were short of a country-wide profile of the burden. For instance, methicillin-resistant *Staphylococcus aureus* (MRSA) was highly prevalent (overall, 46%) among patients (52%), healthcare workers (36%), and the clinical environment (33%) of the burns unit of Mulago National Referral Hospital (NRH) [4]. Further, multidrug-resistant (MDR) Gram-negative rods, including Enterobacterales and *Acinetobacter* spp., were responsible for a large proportion (80%) of puerperal sepsis among postpartum mothers attending a tertiary hospital [5]. Among the Enterobacterales isolated, 82% were extended-spectrum β-lactamase (ESBL) producers, 77% were resistant to ciprofloxacin, and 46% of the *Staphylococcus aureus* isolates were MRSA [5]. 

As Uganda initiated its national AMR surveillance systems in line with the Global Antimicrobial Surveillance System (GLASS), a situational analysis revealed high levels of MDR due to MRSA (2–90%), ESBL (10–75%), and carbapenem-resistant Enterobacterales (CRE, 4–30%), as well as high resistance (>50%) to common antibiotics including penicillin, tetracycline, and cotrimoxazole [6]. Uganda has since made significant progress in setting up its national AMR surveillance system, guided by a National AMR Surveillance Plan (NAP) based on the GLASS manual [7]. Some of the initial surveillance data show that common pathogens including Enterobacterales and non-fermenting Gram-negative bacilli are resistant to third-generation cephalosporins, and there is emerging resistance to carbapenems [7]. For instance, *Escherichia coli* resistance to ceftriaxone, imipenem, and ciprofloxacin was 52.7%, 18.8%, and 52% respectively, while *Klebsiella pneumoniae* resistance to the same antibiotics was 79.7%, 1.6%, and 53.8% respectively [7,8]. This wide perspective of the country’s AMR burden has been enabled by coordinated surveillance guided by the NAP, national governance structures, surveillance documents, and an expanded microbiology laboratories network [7].

Although the current national AMR surveillance data have informed the early AMR surveillance and AMR control efforts, a more comprehensive and well-structured profiling of the AMR burden to tailor the response efforts to the different levels of the health care system is still lacking. This evaluation therefore sought to conduct an extended profiling of the AMR burden and its variation with critical determinants including age, gender, sample types, and healthcare units at selected tertiary hospitals to fill the above gap. This information will be critical in informing and evaluating infection prevention and antimicrobial stewardship interventions aimed at preventing, slowing, and reversing the ascendance of the AMR epidemic in the country. It will also give insights into the performance of the AMR surveillance program and critical areas that need improvement.

## 2. Methodology

**Figure 1:**
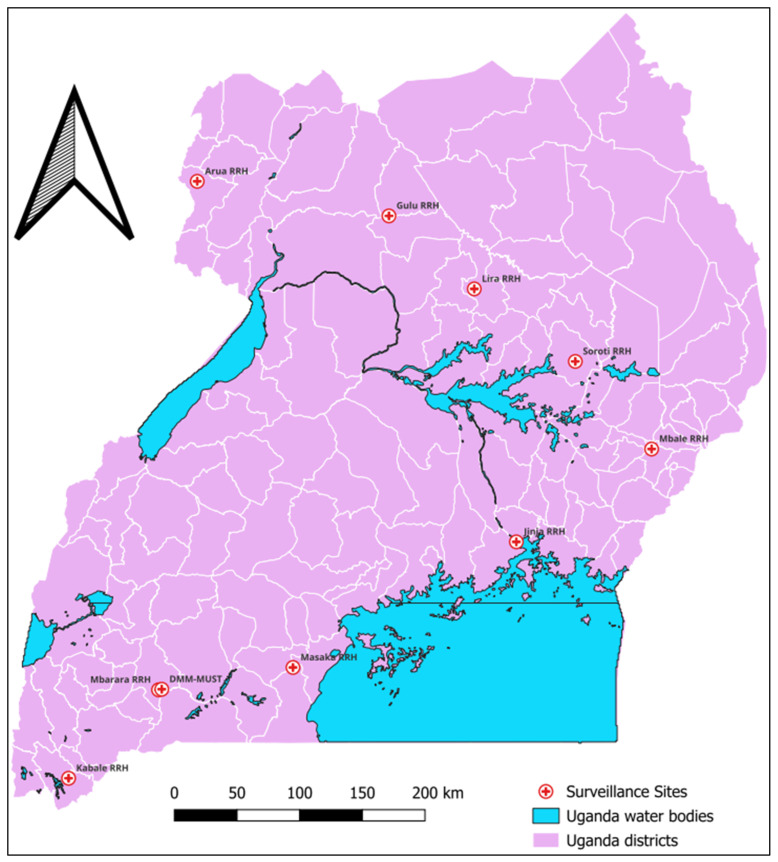
Map showing the AMR surveillance sites that were included in this evaluation.

This was an analysis of the routine laboratory-based surveillance data collected between October 2020 and March 2023 from 10 tertiary hospitals, including nine regional (sub-national) referral hospitals and one national referral hospital in Uganda (Figure 1).

The country has a tiered healthcare system whose base is formed by health centers at the community level, followed by general hospitals, regional referral hospitals (RRH), and national referral hospitals. Regional referral hospitals are the first level, with specialists in the major fields of surgery, internal medicine, obstetrics and gynecology, and pediatrics. The national referral hospitals offer super-specialized healthcare with multiple specialists in different fields. Microbiology laboratory capacity has been gradually developed in the facilities over the past five years. At the time of data collection, all laboratories in the surveillance network were receiving continuous mentors in quality management systems using the strengthening laboratory management towards accreditation (SLMTA) and stepwise laboratory quality improvement process towards accreditation (SLIPTA) approaches. All the labs had applied for SANAS assessment for accreditation (Star 3–5).

Qualified clinical and laboratory teams are deployed to collect and analyze the samples, and these undergo continuous in-service training and mentorship in diagnostic stewardship, AMR surveillance guidelines and protocols, bacteriology techniques, and laboratory quality management systems. Standard laboratory procedures are followed during sample processing. In-house reconstituted culture media are used for primary isolation, including 5% sheep blood agar, MacConkey, and chocolate agar. Bacterial identification was carried out using biochemical methods, including colonial appearance and Gram stain reactions. Culture media is quality controlled for sterility and ability to support growth using recommended American Type Culture Collection (ATCC) reference bacterial strains. Mueller Hinton agar (MHA) media performance and potency of antibiotic discs were assessed using *Escherichia coli* ATCC 25922, *Staphylococcus aureus* ATCC 25923, and *Pseudomonas aeruginosa* ATCC 27853 [9]. Antimicrobial susceptibility testing (AST) was performed using the Kirby-Bauer disc diffusion method according to the updated CLSI M100, 30th, 31st, 32nd, and 33rd editions [10,11,12].

Specimens were collected from patients with various community- or hospital-acquired infections. The analysis included both GLASS priority (urine, blood, stool, urogenital, and cerebrospinal fluid (CSF)) and non-GLASS specimens, including pus and tracheal aspirates. Patient demographic and clinical details were collected using the standardized national microbiology laboratory requisition form, which accompanies all specimens to the laboratory. For purposes of this evaluation, we categorized the hospital units as “General units/wards”, referring to those that provide care to relatively clinically stable patients such as outpatient, medical, surgical, and pediatric, among other wards, and “specialized units/wards”, referring to units that provide care to critically ill patients, including high dependence, intensive care, and dialysis units.

### 2.1. Sample Size

The total number of patients sampled across the surveillance sites during the evaluation period was 14,484, which constituted the sample size for the evaluation. Results from blood, CSF, urine, pus, and tracheal aspirate specimens cultured during the study period were included in the analysis. 

### 2.2. Statistical Analysis

Unique patient identification numbers and results were entered into the WHONET 5.6, AMR data management software [13].

For summary statistics, continuous variables were reported as means with standard deviation (SD) for normally distributed data or median and interquartile range (IQR) for non-normally distributed data. Categorical variables were reported as proportions in terms of frequencies and percentages. To derive the percentage of susceptible isolates for each pathogen-antibiotic pair, the number of susceptible isolates was divided by the total number of isolates tested against each antibiotic and multiplied by 100. Susceptibility was stratified by age, sex, specimen type, and hospital unit. Associations between susceptibility and these variables were established using the Chi Square test. The resulting percentages were reported with respective 95% confidence intervals (CI) and *p*-values. The three-month changes in the trends of susceptibility over the evaluation period were determined using linear regression analysis. Data analysis was performed in StataSE 15, R 4.2.2 software, and Microsoft Excel version 2020. At all levels of comparison, the level of significance was a paired *p*-value < 0.05.

### 2.3. Ethical Statement

The data used in the evaluation are part of the national AMR surveillance data extracted from the electronic laboratory records system as mandated by the Ministry of Health. The evaluation used de-identified aggregate data. There was no interaction with the patients. 

## 3. Results

Table 1 summarizes the demographic characteristics. A total of 95,371 patients presented to the surveillance sites with suspected infections during the evaluation period. Of the 95,371 patients, 14,484 (15%) underwent microbiological testing, of which most, 8002 (55.74%), were female. Of the specimens analyzed, the majority (3279) were urine, followed by blood (3245) and pus (2774). A total of 2754 isolates were recovered, including 1443 (52.4%) from pus, 1035 (37.6%) from urine, 245 (8.9%) from blood, and the rest from CSF, stool, urogenital, tracheal aspirates, and sputum.

Supplementary Table S1 shows the isolate recovery from the different sample types. Overall, *Staphylococcus aureus* was the most common pathogen, contributing 1020 (37%) isolates, followed by *Escherichia* coli, 808 (29.3%) and *Klebsiella* spp., 200 (7.3%). Of the 245 blood isolates, 121 (49.4%) were *Staphylococcus aureus*, 91 (37.1%) were Enterobacterales (*Klebsiella* spp., *Escherichia coli*, and *Enterobacter* spp.), 17 (6.9%) were *Enterococcus* spp., 7 (2.9%) were *Salmonella* spp., 5 (2.04%) were *Acinetobacter* spp., 3 (1.22%) were *Pseudomonas* spp. and 1 (0.4%) was *Streptococcus pneumoniae*.

*Escherichia coli*, 443 (42.8%), was the most common urine isolate, followed by *Staphylococcus aureus*, 299 (28.9%), *Klebsiella* spp., 115 (11.1%), *Enterococcus* spp., 64 (6.2%), *Citrobacter* spp., 52 (5.0%), *Enterobacter* spp., 29 (2.8%), *Pseudomonas* spp. (20, 1.93%), and *Acinetobacter* spp., 5 (0.8%).

The most common pus isolates were Enterobacterales, 697 (48.3%), majorly *Escherichia coli*, 325 (47%), then *Klebsiella* spp., 200 (29%), and *Citrobacter* spp., 120 (17%). Among the Gram-positive pathogens, *Staphylococcus aureus*, were 600 (41.5%), while the *Enterococcus* spp. were 20 (1.9%). The non-fermenting Gram-negative isolates included *Pseudomonas* spp. 69 (6.7%) and *Acinetobacter* spp. 57 (5.5%)). Overall, most of the isolates, 2572 (92%), were ESKAPE-E pathogens. However, from the available information, we could not distinguish between community-acquired infections (CAI) and HAI. 

### 3.1. Susceptibility of Isolates in General and Specialized Units

Susceptibility data are summarized in Table 2 (for general units) and Supplementary Table S2 (for specialized units). For Gram-negatives, data for ampicillin were only available for *Escherichia coli*, showing a remarkably low susceptibility of 4% for isolates from specialized clinics and 8% for general clinics. *Escherichia coli* susceptibility to the combination amoxycillin/clavulanic acid was much lower for specialized clinics (20%) compared to general clinics (56%).

Ceftriaxone, the only third-generation cephalosporin analyzed, also had very low susceptibility, ranging from 28% for *Escherichia coli* to 42% for ‘other Enterobacterales’ in general clinics, but did not remarkably differ from that observed in the specialized clinics. At 19%, meropenem susceptibility was remarkably low for *Acinetobacter* spp. isolates from specialized clinics. Notably, reduced susceptibility to meropenem was also observed for *Klebsiella* spp. isolates from the specialized clinics (70%).

Gentamicin, the only aminoglycoside analyzed, had susceptibilities just below 60% for *Escherichia coli* and *Klebsiella* spp. but higher at 71% and 65% for other Enterobacterales and *Pseudomonas* spp., respectively. Susceptibility to ciprofloxacin, representing the second-generation quinolones, showed low or modest susceptibilities ranging from 36% for *Escherichia*. *coli* to 60% for *Pseudomonas* spp.

Among *Staphylococcus aureus*, only 44% were methicillin-susceptible, indicating a remarkable 56% MRSA prevalence. Susceptibility to erythromycin was also very low, at 34%. There were no data available for vancomycin. Among the enterococci, 66% were susceptible to ampicillin and 72% to vancomycin. 

### 3.2. Antibiotic Susceptibility Variation by Hospital Units

Table 3 presents the distribution of bacterial susceptibility across various hospital units. There were significant variations in susceptibility to all tested antibiotics (*p* = 0.000–0.025) across the different hospital units, except for ciprofloxacin (*p* = 0.888), chloramphenicol (*p* = 0.109), meropenem (*p* = 0.136), and vancomycin (*p* = 0.64). The hospital units included the emergence department, gynecology ward, HIV clinic, maternity, outpatients’ department, pediatric ward, and surgical ward. Notably, the susceptibility observed in the HIV clinic was higher than that in other units. However, the number of isolates from the HIV clinic was few (<30).

### 3.3. Antibiotic Susceptibility Variation by Age Group

Table 4 presents the distribution of bacterial susceptibility across different age groups, revealing significant variations in susceptibility patterns for various antibiotics across different age cohorts. Among individuals aged 5–24 years, the susceptibility to ampicillin resistance was 8% lower [OR = 0.54, CI = 0.33–0.88, *p* = 0.013] compared to those aged >65 years. Moreover, across all age groups, ampicillin susceptibility remained consistently low, below 20% for most age groups. In the 25–44 age group, there was an 8% lower susceptibility to ciprofloxacin [OR = 1.4, CI = 1.072–1.835, *p* = 0.014] compared to the >65 age group. Additionally, the 0–4 age group exhibited an 8% higher ciprofloxacin susceptibility [OR = 0.73, CI = 0.532–0.993, *p* = 0.045] compared to the >65 age group. Overall, the susceptibility to ciprofloxacin was below 60%. 

The age groups of 25–44 and 45–64 demonstrated an 11% [OR = 1.66, CI = 1.155–2.377, *p* = 0.006] and 10% [OR = 1.54, CI = 1.042–2.274, *p* = 0.030] lower chloramphenicol susceptibility, respectively, in comparison to the >65 age group. In contrast, there was remarkably higher methicillin susceptibility among *Staphylococcus aureus* isolates from the 25–44 age group (77% vs. 20%) [OR = 0.23, CI = 0.061–0.885, *p* = 0.032] compared to the >65 age group, indicating lower MRSA in the 25–44 age group. Susceptibility to third-generation cephalosporins, amoxicillin clavulanic acid, and carbapenems did not vary significantly across age groups.

### 3.4. Antibiotic Susceptibility Variation by Sex

Supplementary Table S3 displays the distribution of antimicrobial susceptibility by sex, revealing significantly higher susceptibility to erythromycin among males compared to females [OR = 0.75, CI = 0.595–0.941, *p* = 0.013]. Susceptibility to other antibiotics did not vary by sex.

### 3.5. Antibiotic Susceptibility Variation by Sample Type

Table 5 compares the susceptibility profiles of isolates from various specimen types. For *Escherichia coli*, differences were noted for ciprofloxacin, chloramphenicol, and ceftriaxone. Ciprofloxacin susceptibilities were higher in blood (57%) compared to urine (39%) or pus (28%), *p*-value of 0.005. Similarly, higher susceptibility was observed for ceftriaxone for isolates from blood (44%) compared to urine (34%) or pus (14%), *p* = 0.002. For chloramphenicol, on the other hand, susceptibilities were highest for pus (75%) and urine (72%) and lowest for blood (33%). For *Klebsiella* spp., blood isolates demonstrated lower susceptibilities for chloramphenicol than those for urine or pus (41% vs. 77% vs. 39%, respectively, *p* = 0.008). This was also the case for ceftriaxone, with susceptibilities of 19% for blood, 56% for urine, and 28% for pus (*p* = 0.006). In that case of gentamicin, susceptibilities were lower for blood (39%) and pus (42%) than urine (60%), *p* = 0.049.

### 3.6. Antibiotic Susceptibility Trends

**Figure 2:**
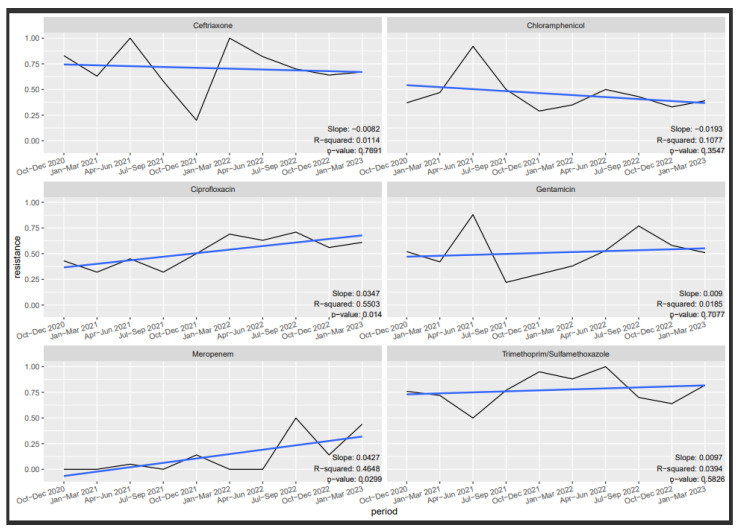
Trend in *Klebsiella* spp. resistance.

**Figure 3:**
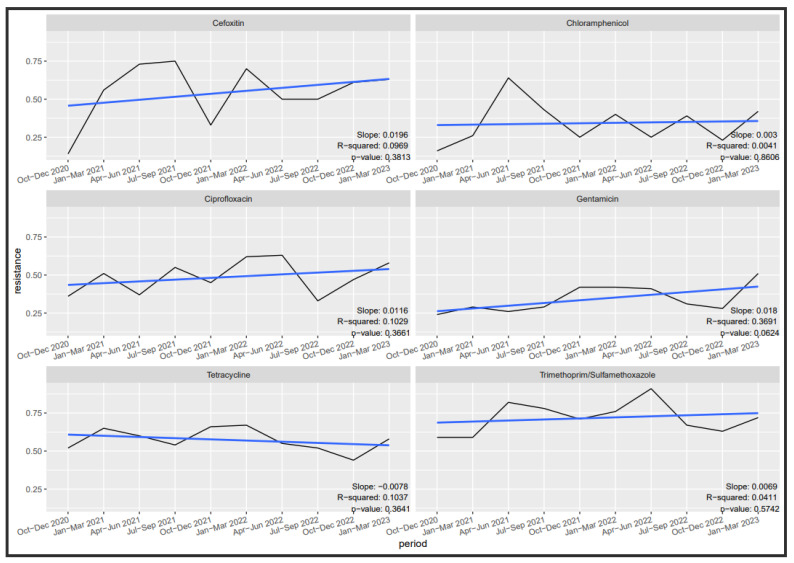
Trend in *Staphylococcus aureus* resistance.

**Figure 4:**
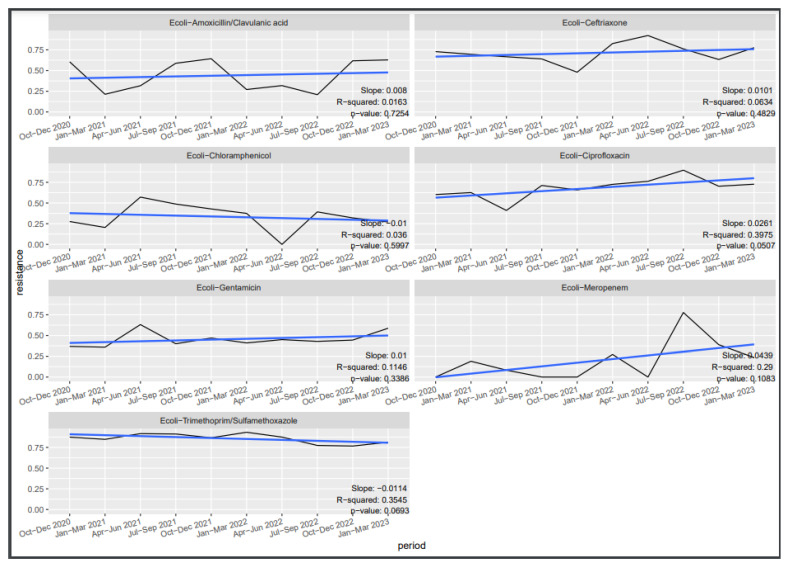
Trends in *E*. *coli* resistance.

Figure 2, Figure 3 and Figure 4 depict the trends in susceptibilities observed during the evaluation period. Specifically, we investigated the quarterly susceptibility trends of *Klebsiella* spp., *Staphylococcus aureus*, and *Escherichia coli* isolates to various antibiotics.

Notably, significant quarterly variation was observed for *Klebsiella* spp. susceptibility to ciprofloxacin and meropenem, indicating notable changes in susceptibility over time (*p* = 0.01 and 0.029, respectively). Ciprofloxacin susceptibility displayed a negative trend (slope = 0.0347), with approximately a 55% decrease in susceptibility by quarter (R-squared = 0.5503). Similarly, meropenem exhibited a negative trend (slope = 0.0427), with approximately a 47% decrease in susceptibility by quarter (R-squared = 0.4648). However, no significant variation was observed for ceftriaxone, chloramphenicol, gentamicin, and cotrimoxazole.

For *Staphylococcus aureus*, only gentamicin susceptibility demonstrated a marginal variation by quarters (*p*-value = 0.062). Gentamicin exhibited a decreasing trend (slope = 0.018), with approximately a 36.91% decrease in susceptibility by quarter (R-squared = 0.3691). There was no significant variation in susceptibility by quarter for cefoxitin, ciprofloxacin, tetracycline, chloramphenicol, and cotrimoxazole.

For *Escherichia coli*, varying susceptibility by quarter was observed. Susceptibility to ciprofloxacin demonstrated a significant negative trend over the quarters (slope = 0.0262, R-squared = 0.3967, *p* = 0.05), while cotrimoxazole also exhibited a marginally significant negative trend (slope = −0.0119, R-squared = 0.3832, *p* = 0.056). No significant variation was observed for the remaining antibiotics: amoxicillin, meropenem, ceftriaxone, and gentamicin.

## 4. Discussion

We provide an extensive evaluation of AMR in Uganda, focusing primarily on the most isolated bacteria: *Escherichia coli*, *Klebsiella* spp., *Acinetobacter* spp., *Staph*. *aureus*, and *Enterococcus* spp. These ESKAPE organisms contributed 92% of the isolates, with susceptibility to specific antibiotics varying significantly among sample types and healthcare units. Remarkably reduced susceptibility was observed in all categories of the antibiotics, including the Access and Watch groups (AWaRe classification), with worryingly low susceptibility in the Watch category, more so in the specialized clinics. Similarly, resistance (reduction in susceptibility) was spread across all age groups, with resistance to certain antibiotics like ampicillin, ciprofloxacin, chloramphenicol, gentamycin, and methicillin varying significantly in certain age groups. Over the evaluation period, resistance was mostly linear except for ciprofloxacin, meropenem, and gentamycin, which showed a positive trend. Similarly, co-trimoxazole showed a slight positive trend for *Klebsiella* spp. and *Staph*. *aureus* with no significant change for *Escherichia coli*. A pronounced spike was observed in the *E*. *coli*, *Klebsiella* spp., and *Staphylococcus aureus* resistance between April and June 2021, the same timing of the COVID-19 pandemic peak. Notably, resistance against many antibiotics, particularly ampicillin and cotrimoxazole, was persistently high throughout the evaluation period.

Antimicrobial resistance to commonly prescribed antibiotics is widespread in the East African region, especially by Gram-negative bacteria [14]. Earlier studies heralded AMR as an emerging threat in Uganda: Seni et al. found alarmingly high levels of MDR pathogens, particularly ESBL and MRSA, in surgical site infections at Mulago hospital as early as 2013 [15]. Moreover, the epidemic was already occurring in the community; MRSA and ESBLs were highly prevalent in community acquired pyogenic abscesses presenting to Mulago hospital [16]. Our evaluation demonstrated that there has been an increase in AMR compared to these earlier evaluations of surveillance data before and after the constitution of the NAP-AMR, both in terms of AMR prevalence and spread [6,7,8]. The Gram-negatives exhibited high resistance to ceftriaxone, imipenem, ciprofloxacin, gentamycin, and chloramphenicol, and the high amoxicillin clavulanic acid resistance in *Escherichia coli*, presumably indicating a role for AmpC beta lactamases. Similarly, the high resistance to imipenem/meropenem by *Escherichia coli*, *Klebsiella* spp., and *Acinetobacter* suggested a high presence of carbapenamase producers. However, laboratory confirmation for ESBL and carbapenamase-producing organisms (CPO) was not done. The observed increase suggests that the stewardship actions being used are not having the desired effect, which requires immediate attention.

Among the Gram-positives, the very high prevalence of MRSA at 56% was particularly notable and comparable to the 53% found by Pius [17] from skin and soft tissue infections in western Uganda. Similarly, high rates of MRSA have been reported in Kenya (53.4%) [18] and Tanzania (43.3%) [19], suggesting that MRSA is endemic in Uganda and other East African countries. MRSA isolation from community- and hospital-acquired infections suggests continued emergence and transmission in both contexts, necessitating IPC and the promotion of rational antibiotic usage in both. Among the enterococci, 66% were still susceptible to ampicillin but, worryingly, vancomycin resistance was up to 24%. One study in Kenya identified no vancomycin resistance among clinical *Enterococcus* isolates [20]. The World Health Organization listed the carbapenemase producers, ESBL, MRSA, and VRE, as critical and high-priority threats requiring urgent attention [21]. The prevalence of the above resistance phenotypes was higher in specialized healthcare units like the ICU compared to the general units. Specialized units are characterized by extensive use of broad-spectrum antibiotics, invasive procedures, and the handling of patients with severe infections, which are predisposing factors for AMR [22,23]. Moreover, resistance to all antibiotics in this evaluation significantly varied among the different healthcare units of the hospitals except for ciprofloxacin, chloramphenicol, cefixime, cefepime, and vancomycin. Therefore, context-specific interventions in addition to universal IPC and stewardship interventions are needed.

In this evaluation, ampicillin, ciprofloxacin, chloramphenicol, gentamycin, and methicillin resistance were significantly higher in certain age groups compared to the >65 age group. This indicates that age might be an independent risk factor for pathogens developing resistance to certain antibiotics. Hossain et al. also observed an age effect in urinary tract *Escherichia coli* resistance to antibiotic therapy [24]. *Escherichia coli* resistance to amikacin, nitrofurantoin, and colistin was lower in the younger age groups compared to the >60 age group [24]. Moreover, aging-related physiological, metabolic, and immunological changes are associated with specific changes in different body site microbiota, e.g., skin microbiota changed due to changes in skin sebum [25]. The human microbiota has been deemed a reservoir of AMR due to its wide microbial genetic diversity, ecosystem for genetic exchange, and constant exposure to AMR determinants [26]. This emphasizes that surveillance of antimicrobial resistance genes (ARG) in the human microbiota can be a source of information for AMR control strategies. On the other hand, there was less variation in AMR by sex, with only resistance to erythromycin being significantly lower in men than women. This contrasts Brandl et al. findings from the German national surveillance data, where men had a twofold higher incidence of resistant infections (2.3 for MRSA, 2.2 for carbapenem-resistant *Acinetobacter baumannii*, CRAB, and 1.7 for carbapenem-resistant *Enterobacterales*, CRE) and colonization compared to women [27]. Men are favored in accessing health services because they hold the decision-making power and control of resources, factors that give them access to antimicrobials, a risk of AMR [28]. Women, on the other hand, are more likely to attend local and free health facilities, which might prescribe antibiotics only when needed or when available [29]. These findings show that gender-related effects on AMR are critical in efforts towards the control of AMR, which need to be urgently explored.

Resistance to several antibiotics including, ciprofloxacin, chloramphenicol, ceftriaxone, gentamicin, and erythromycin, significantly differed among the specimen types. This finding may have implications for empirical therapy for different infectious syndromes, as the infection may be due to microorganisms of the same species but different strains [30,31]. However, few prior research efforts have focused on the effect of specimen type on AMR. Studies by Mwansa et al. showed that *Staphylococcus aureus* resistance to tetracycline was influenced by specimen type, being lower in urine compared to blood [32]. Likewise, *Escherichia coli* isolated from sputum had higher resistance to common antibiotics compared to those isolated from blood and urine, except for urine, where *Escherichia coli* was more resistant to fluoroquinolones than that from sputum [33]. Similarly, *Salmonella enterica* isolated from urine, blood, and stool had differing resistance to cephems, macrolides, phenicols, tetracyclines, and quinolones [30]. The largest difference was observed with quinolones, to which the isolates from blood were less susceptible compared to those from urine or feces [30]. It is plausible that the chemical and immunological properties of the different specimens that affect bacterial yield from the different specimens [34] might also alter the organism’s response to antibiotic therapy. The GLASS also set priority specimens for surveillance based on infections of critical body systems that were showing alarming resistance to last resort antibiotics [35]. The variation of resistance by specimen type observed in these studies emphasizes that for antibiograms to effectively inform AMR control, they need to be context-specific, including specimen type, organism, age, and others whenever possible.

We provide the first evaluation of resistance trends in bacteria predominantly isolated in Uganda. Positive trends in antibiotic resistance were noted for ciprofloxacin and meropenem for *Klebsiella* spp., gentamycin for *Staphylococcus aureus*, and ciprofloxacin for *Escherichia coli*, while cotrimoxazole showed a slight negative trend for *Escherichia coli*. The antibiotic resistance of most bacteria to the other antibiotics remained persistently above 30%, with none showing any negative trend. Selection pressure due to irrational use of antibiotics and the cloning of the selected resistant isolates likely underlie this persistently high and increasing trend in resistance against certain antibiotics [36]. Notable was a pronounced spike in *Escherichia coli* resistance to amoxicillin clavulanic acid and gentamycin; *Klebsiella* spp. resistance to ceftriaxone, chloramphenicol, and gentamycin; and *Staph*. *aureus* resistance to cefoxitin and chloramphenicol during the COVID-19 pandemic peak. The Center for Disease Control reported a 15% increase in AMR between 2019 and 2020, largely driven by a breakdown in infection prevention measures and antibiotic prescriptions for supportive treatment of COVID-19 and bacterial co-infections [37]. The increasing trends and persistently high resistance have implications for the empirical treatment of infectious syndromes [38], which is the commonest approach to treating bacterial infection in Uganda. Antimicrobial stewardship implementation strategies are urgently required to reverse the observed, but these would be more effective if they were targeted at specific situations or antimicrobials [39].

The greatest strength of our evaluation is that, unlike prior evaluations of AMR surveillance in Uganda, our evaluation presents a wide interrogation of the AMR surveillance data and considers how some critical parameters like age, sex, specimen types, and healthcare unit may influence the AMR surveillance outlook. This information is very critical to shaping Uganda’s AMR surveillance efforts focused and effective. The evaluation was limited by the range of antibiotics tested and the range of pathogens considered, focusing on *Escherichia coli*, *Klebsiella* spp., *Acinetobacter* spp., *Staph*. *aureus*, and *Enterococcus* spp., which had large representative numbers for analysis.

## 5. Conclusions

We noted consistently high resistance across all WHO AWaRe categorizations of antibiotics, with increasing trends for certain antibiotics and significant variations by age, sex, specimen type, and hospital unit. The findings emphasize the necessity of transitioning to surveillance combined with infection prevention and effective antimicrobial stewardship strategies targeted at the critical drivers of AMR so as to reverse the ascending trends of AMR. Further, gender issues relating to AMR need urgent investigation, while molecular and genetic surveillance techniques, particularly those focused on ARG in the body microbiota, would improve stewardship interventions.

## Figures and Tables

**Table 1:** Demographics characteristics.

Variable	Levels	Number of Specimens	Percentage of Total
Site	Jinja RRH	2821	19.48%
	DMM MUST	2615	18.05%
	Mbarara RRH	2384	16.46%
	Kabale RRH	1881	12.99%
	Mbale RRH	1487	10.27%
	Arua RRH	1039	7.17%
	Lira RRH	820	5.66%
	Gulu RRH	656	4.53%
	Masaka RRH	538	3.71%
	Soroti RRH	243	1.68%
Sex	Female	8002	55.74%
	Male	6355	44.26%
Age (Years)	25–44	4902	33.84%
	5–24	3976	27.45%
	0–4	2532	17.48%
	45–64	2075	14.33%
	65 and above	999	6.90%
Sample type	Urine	3279	25.29%
	Blood	3245	25.03%
	Pus Swab	2774	21.39%
	Urogenital swabs	1781	13.73%
	Cerebral Spinal Fluid	881	6.79%
	Sputum	564	4.35%
	Stool	407	3.14%
	Tracheal aspirate	36	0.28%
Quarter	October–December 2020	1936	13.37%
	January–March 2021	2159	14.91%
	April–June 2021	2093	14.45%
	July–September 2021	1881	12.99%
	October–December 2021	1366	9.43%
	January–March 2022	1120	7.73%
	April–June 2022	667	4.61%
	July–September 2022	677	4.67%
	October–December 2022	852	5.88%
	January–March 2023	1733	11.96%
Department	Outpatient department	5282	45.31%
	Paediatric ward	2141	18.37%
	Medical ward	1365	11.71%
	Surgical ward	885	7.59%
	Gynaecology ward	547	4.69%
	Emergency ward	537	4.61%
	Orthopaedic department	266	2.28%
	Maternity ward	168	1.44%
	Private ward	134	1.15%
	Acute Care Unit	133	1.14%
	Intensive care unit	106	0.91%
	HIV clinic	94	0.81%

**Table 2:** Antibiotic susceptibility in general clinics.

Antibiotic	Gram-Negative Isolates					Gram-Positive Isolates	
	Enterobacterales			Non Enterobacterales			
	*E*. *coli* n (%S)	*Klebsiella* spp. n (%S)	Others n (%S)	*Acinetobacter* spp. n (%S)	*Pseudomonas* spp. n (%S)	*Staphylococcus aureus* n (%S)	*Enterococcus* spp. n (%S)
Ampicillin	607 (8%)					79 (22%)	70 (66%)
Amoxicillin/Clavulanic acid	463 (56%)		11 (82%)				
Ciprofloxacin	650 (36%)	299 (55%)	220 (58%)	69 (54%)	73 (60%)	799 (51%)	86 (51%)
Chloramphenicol	411 (67%)	179 (56%)	124 (39%)			631 (68%)	49 (59%)
Ceftriaxone	375 (28%)	226 (38%)	155 (42%)	31 (16%)			
Meropenem	172 (81%)	86 (90%)	77 (75%)	20 (55%)	19 (63%)		
Imipenem	629 (89%)	365 (90%)	201 (77%)	59 (66%)	94 (87%)		
Trimethoprim	466 (15%)	275 (21%)	167 (36%)	40 (20%)		597 (32%)	
Gentamicin	597 (57%)	278 (54%)	192 (71%)	57 (53%)	60 (65%)	718 (66%)	
Erythromycin						748 (34%)	
Vancomycin							86 (72%)
Cefoxitin (MRSA Screen)						172 (44%)	

**Table 3:** Susceptibility variation by hospital units.

Antibiotic	Emergency n (%S)	Gynaecology n (%S)	HIV Clinic n (%S)	Maternity n (%S)	Medical n (%S)	OPD n (%S)	Paediatrics n (%S)	Specialized n (%S)	Surgical n (%S)	*p*-Value
Ampicillin	38 (18%)	83 (14%)	12 * (33%)	27 * (7%)	150 (13%)	608 (17%)	84 (10%)	24 * (4%)	230 (7%)	0
Amoxicillin/Clavulanic acid	23 * (35%)	71 (45%)	9 * (56%)	16 * (50%)	121 (42%)	434 (54%)	70 (41%)	9 * (44%)	188 (47%)	0.007
Ciprofloxacin	80 (46%)	157 (44%)	17 * (33%)	47 (36%)	251 (46%)	886 (48%)	195 (56%)	35 (49%)	416 (44%)	0.888
Chloramphenicol	32 (56%)	93 (61%)	11 * (82%)	42 (55%)	137 (66%)	622 (60%)	139 (69%)	24 * (50%)	318 (58%)	0.109
Ceftriaxone	18 * (17%)	58 (21%)	7 * (43%)	8 * (25%)	115 (27%)	315 (57%)	76 (26%)	14 * (21%)	196 (22%)	0
Meropenem	8 * (87%)	27 * (74%)		5 * (40%)	55 (89%)	161 (86%)	19 * (79%)	2 * (50%)	67 (67%)	0.136
Imipenem	51 (90%)	96 (77%)	12 * (92%)	27 * (89%)	194 (89%)	627 (90%)	112 (85%)	32 (75%)	249 (84%)	0.025
Trimethoprim/Sulfamethoxazole	31 (6%)	96 (19%)	13 * (8%)	48 (21%)	173 (20%)	694 (24%)	161 (25%)	17 * (24%)	240 (21%)	0
Gentamicin	61 (70%)	126 (44%)	18 * (67%)	50 (56%)	193 (64%)	767 (65%)	153 (51%)	23 * (61%)	400 (55%)	0
Erythromycin	24 * (33%)	57 (23%)	12 * (33%)	24 * (25%)	90 (41%)	527 (26%)	130 (32%)	11 * (27%)	157 (45%)	0
Vancomycin	5 * (20%)	26 * (46%)	1 * (0%)	3 * (67%)	48 (42%)	249 (36%)	28 * (61%)	6 * (67%)	24 * (21%)	0.64
Oxacillin/Methicillin	10 * (50%)	9 * (44%)	1 * (0%)	2 * (50%)	17 * (53%)	82 (44%)	38 (47%)	1 * (0%)	37 (59%)	0

* Bacterial isolates less than 30, which limits our ability to use the data to inform patient care or any stewardship or policy interventions.

**Table 4:** Susceptibility variation by age groups.

Antibiotic Name	Age Groups	Susceptibility	OR [95% CI]	*p*-Values
Ampicillin	0–4	173 (13%)	0.86 [0.468–1.588]	0.634
	5–9	41 (21%)	0.45 [0.191–1.043]	0.063
	10–14	40 (22%)	0.43 [0.185–1.013]	0.054
	15–25	294 (18%)	0.57 [0.343–0.952]	0.032
	25–44	556 (12%)	0.86 [0.528–1.393]	0.536
	45–64	332 (14%)	0.74 [0.444–1.246]	0.26
	65 and above	224 (11%)	Ref	
Amoxiclav	0–4	117 (47%)	1.24 [0.773–1.985]	0.374
	5–9	34 (58%)	0.77 [0.365–1.622]	0.491
	10–14	31 (45%)	1.33 [0.619–2.878]	0.462
	15–25	205 (55%)	0.88 [0.583–1.319]	0.529
	25–44	386 (46%)	1.24 [0.867–1.786]	0.235
	45–64	240 (46%)	1.28 [0.861–1.893]	0.224
	65 and above	170 (52%)	Ref	
Ciprofloxacin	0–4	377 (58%)	0.73 [0.532–0.993]	0.045
	5–9	67 (62%)	0.6 [0.346–1.038]	0.068
	10–14	91 (74%)	0.34 [0.201–0.578]	0.000
	15–25	567 (47%)	1.12 [0.837–1.489]	0.456
	25–44	964 (41%)	1.4 [1.072–1.835]	0.014
	45–64	505 (45%)	1.19 [0.883–1.591]	0.257
	65 and above	275 (50%)	Ref	
Chloramphenicol	0–4	253 (64%)	1.15 [0.757–1.733]	0.520
	5–9	55 (69%)	0.94 [0.49–1.822]	0.865
	10–14	50 (68%)	0.99 [0.505–1.954]	0.985
	15–25	397 (65%)	1.09 [0.74–1.598]	0.668
	25–44	614 (56%)	1.66 [1.155–2.377]	0.006
	45–64	332 (57%)	1.54 [1.042–2.274]	0.030
	65 and above	168 (67%)	Ref	
Ceftriaxone	0–4	105 (27%)	1.64 [0.936–2.881]	0.084
	5–9	34 (38%)	1.01 [0.463–2.213]	0.976
	10–14	29 (58%)	0.44 [0.194–1.008]	0.052
	15–25	179 (34%)	1.21 [0.752–1.955]	0.430
	25–44	337 (38%)	1 [0.652–1.527]	0.992
	45–64	216 (31%)	1.36 [0.857–2.169]	0.190
	65 and above	122 (38%)	Ref	
Meropenem	0–4	53 (77%)	2.2 [0.825–5.842]	0.115
	5–9	15 (86%)	1.15 [0.219–6.077]	0.866
	10–14	10 (80%)	1.88 [0.337–10.431]	0.473
	15–25	74 (77%)	2.24 [0.896–5.586]	0.085
	25–44	159 (80%)	1.82 [0.788–4.189]	0.161
	45–64	90 (82%)	1.62 [0.65–4.047]	0.300
	65 and above	68 (88%)	Ref	
Imipenem	0–4	197 (82%)	1.73 [0.991–3.011]	0.054
	5–9	43 (86%)	1.34 [0.516–3.497]	0.546
	10–14	61 (85%)	1.43 [0.631–3.255]	0.390
	15–25	314 (85%)	1.35 [0.8–2.277]	0.262
	25–44	596 (85%)	1.36 [0.845–2.184]	0.206
	45–64	364 (84%)	1.51 [0.91–2.49]	0.111
	65 and above	232 (89%)	Ref	
Trimethoprim Sulphamethoxazole	0–4	267 (25%)	0.79 [0.512–1.223]	0.292
	5–9	63 (31%)	0.58 [0.31–1.09]	0.091
	10–14	69 (34%)	0.51 [0.279–0.923]	0.026
	15–25	360 (22%)	0.93 [0.613–1.415]	0.739
	25–44	670 (22%)	0.94 [0.639–1.375]	0.741
	45–64	331 (25%)	0.81 [0.532–1.228]	0.318
	65 and above	202 (21%)	Ref	
Gentamicin	0–4	307 (51%)	1.7 [1.199–2.402]	0.003
	5–9	57 (73%)	0.63 [0.332–1.211]	0.168
	10–14	70 (80%)	0.44 [0.233–0.845]	0.013
	15–25	494 (60%)	1.15 [0.833–1.584]	0.398
	25–44	832 (58%)	1.27 [0.942–1.715]	0.116
	45–64	420 (59%)	1.22 [0.878–1.696]	0.237
	65 and above	236 (63%)	Ref	
Erythromycin	0–4	215 (31%)	0.99 [0.577–1.695]	0.969
	5–9	49 (44%)	0.56 [0.272–1.159]	0.118
	10–14	60 (50%)	0.46 [0.232–0.904]	0.024
	15–25	300 (35%)	0.85 [0.509–1.42]	0.535
	25–44	477 (28%)	1.14 [0.691–1.866]	0.616
	45–64	196 (30%)	1.04 [0.6–1.793]	0.896
	65 and above	86 (31%)	Ref	
Vancomycin	0–4	59 (44%)	0.71 [0.33–1.547]	0.393
	5–9	17 (47%)	0.63 [0.208–1.927]	0.421
	10–14	15 (40%)	0.84 [0.258–2.755]	0.778
	15–25	72 (37%)	0.94 [0.443–1.983]	0.866
	25–44	150 (40%)	0.82 [0.423–1.593]	0.559
	45–64	97 (38%)	0.91 [0.449–1.852]	0.799
	65 and above	50 (36%)	Ref	
Oxacillin/Methicillin	0–4	55 (38%)	0.4 [0.102–1.604]	0.198
	5–9	9 (55%)	0.2 [0.032–1.24]	0.084
	10–14	18 (77%)	0.07 [0.013–0.385]	0.002
	15–25	52 (42%)	0.34 [0.086–1.355]	0.126
	25–44	69 (46%)	0.29 [0.075–1.116]	0.072
	45–64	35 (45%)	0.3 [0.071–1.24]	0.096
	65 and above	15 (20%)	Ref	

OR denotes odds ratios, and 95% CI denotes the 95% confidence interval obtained by fitting a logistic regression model. *p*-values < 0.05 were considered statistically significant.

**Table 5:** AMR variation by sample type in general clinics.

Antibiotic	Gram-Negatives												Gram-Positives							
	*Acinetobacter* spp.				*Escherichia coli*				*Klebsiella* spp.				*Staphylococcus aureus*				*Enterococcus* spp.			
	Blood	Urine	Pus	*p*-Value	Blood	Urine	Pus	*p*-Values	Blood	Urine	Pus	*p*-Values	Blood	Urine	Pus	*p*-Value	Blood	Urine	Pus	*p*-Value
	n (%S)	n (%S)	n (%S)		n (%S)	n (%S)	n (%S)		n (%S)	n (%S)	n (%S)		n (%S)	n (%S)	n (%S)		n (%R)	n (%S)	n (%S)	
Ampicillin	NA	NA	NA		20 (20%)	283 (9%)	194 (5%)	0.088	NA	NA	NA		NA	NA	NA	NA	14 (36%)	27 (67%)	8 (87%)	0.046
Amoxicillin/Clavulanic acid	NA	NA	NA		21 (57%)	216 (53%)	147 (56%)	0.705	22 (36%)	55 (44%)	84 (42%)		NA	NA	NA		NA	NA	NA	
Ciprofloxacin	2 (100%)	5 (40%)	44 (57%)	0.047	23 (57%)	256 (39%)	246 (28%)	0.005	29 (41%)	50 (62%)	126 (49%)	0.121	82 (62%)	153 (37%)	407 (62%)	0.000	13 (15%)	39 (64%)	13 (31%)	0.004
Chloramphenicol	NA	NA	NA		12 (33%)	133 (72%)	150 (75%)	0.021	17 (41%)	30 (77%)	89 (39%)	0.008	60 (77%)	128 (58%)	309 (75%)	0.005	12 (67%)	17 (59%)	10 (70%)	0.689
Ceftriaxone					16 (44%)	149 (34%)	133 (14%)	0.002	27 (19%)	45 (56%)	75 (28%)	0.006	NA	NA	NA		NA	NA	NA	
Meropenem		4 (50%)	13 (54%)		6 (83%)	62 (85%)	63 (79%)	0.902	5 (80%)	16 (94%)	34 (88%)	0.636	NA	NA	NA		NA	NA	NA	
Imipenem	5 (80%)	3 (100%)	34 (56%)	0.765	26 (81%)	314 (89%)	200 (86%)	0.302	28 (100%)	78 (91%)	121 (83%)	0.088	NA	NA	NA		NA	NA	NA	
Trimethoprim/Sulfamethoxazole	3 (33%)	1 (100%)	23 (13%)	0.144	15 (27%)	201 (10%)	162 (14%)	0.33	27 (26%)	65 (22%)	84 (15%)	0.473	58 (28%)	135 (21%)	282 (42%)	0.000	NA	NA	NA	
Gentamicin	2 (100%)	5 (40%)	35 (54%)	0.47	18 (61%)	230 (58%)	223 (49%)	0.171	31 (39%)	45 (60%)	109 (42%)	0.049	69 (58%)	142 (61%)	350 (71%)	0.058	NA	NA	NA	
Piperacillin						1 (100%)	1 (0%)				2 (50%)		NA	NA	NA		NA	NA	NA	
Erythromycin	NA	NA	NA		NA	NA	NA		NA	NA	NA		70 (46%)	179 (23%)	352 (41%)	0.000	NA	NA	NA	
Vancomycin	NA	NA	NA		NA	NA			NA	NA	NA		NA	NA	NA		16 (81%)	38 (74%)	12 (58%)	0.440
Oxacillin/ Methicillin	NA	NA	NA		NA	NA			NA	NA	NA		17 (47%)	27 (26%)	99 (51%)	0.123	NA	NA	NA	

## Data Availability

The datasets presented in this article are not readily available because the data is a preserve of the Ministry of Health. Requests to access the datasets should be directed to the Director General of Curative services, Ministry of Health.

## Supplementary Materials

The following supporting information can be downloaded at: https://www.mdpi.com/article/10.3390/tropicalmed9040077/s1. Table S1 Isolate recovery from the different specimen; Table S2: Isolate recovery from the different specimen; Table S3: AMR variation by gender.

## Abbreviations

AMR	Antimicrobial Resistance
AWaRe	Access Watch Reserve
AST	Antimicrobial Susceptibility Testing
GLASS	Global Antimicrobial Resistance and Use Surveillance System
CRE	Carbapenem Resistant Enterobacterales
ESBL	Extended Spectrum Beta Lactamases
MDR	Multi-Drug Resistance
MRSA	Methicillin Resistant Staphylococcus Aureus
NAP	National Action Plan
SLMTA	Strengthening Laboratory Management Towards
Accreditation	
SLIPTA	Laboratory Quality Improvement Process Towards
VRE	Vancomycin Resistant Enterococcus
