# Supplementary material for: Characterization of Antibiotic Resistance in Select Tertiary Hospitals in Uganda: An Evaluation of 2020 to 2023 Routine Surveillance Data
Source: Trop Med Infect Dis. 2024 Apr 5;9(4):77. doi: 10.3390/tropicalmed9040077 (PMC11053536; doi:10.3390/tropicalmed9040077)
Supplement: Supplementary file 1 [file tropicalmed-09-00077-s001.zip › tropicalmed-2865929-supplementary.pdf]

Supplementary Materials: Characterization of Antibiotic Resistance in Select Tertiary Hospitals in Uganda: An Evaluation of 2020 to 2023 Routine Surveillance Data

Table S1. Isolate recovery from the different specimen

	Isolates Recovered by Specimen					
**Isolates**	**Blood**	**Urine**	**Stool**	**Urogenital**	**CSF**	**Pus Swab**
Acinetobacter spp.	5	8				57
Shigella spp.			7			
Salmonella spp.	7					
E. coli	37	443			3	325
Klebsiella spp.	44	115				200
Citrobacter spp.		52				120
Proteus spp.		1				14
Pseudomonas spp.	3	20				69
Enterobacter spp.	10	29				38
S. pneumoniae.	1				4	
Staphylococcus aureus.	121	299				600
Enterococcus spp.	17	64				20
Neisseria gonorrhea		4		17		
Total	245	1,035	7	17	7	1,443

Table S2. General antibiotic resistance in specialized clinics

**Antibiotic**	**Gram-Negative Isolates**					**Gram-Positive Isolates**	
	**Enterobacterales**			**Non Enterobacterales**			
	E. coli **n(%S)**	***Klebsiella*** **spp. n(%S)**	**Others n(%S)**	***Acinetobacter*** **spp. n(%S)**	***Pseudomonas*** **spp. n(%S)**	***Staphylococcus aureus*** **n(%S)**	***Enterococcus*** **spp. n(%S)**
Ampicillin	113 (4%)					26 (0%)	30 (43%)
Amoxicillin/Clavulanic acid	122 (20%)						
Ciprofloxacin	126 (22%)	123 (31%)	66 (39%)	106 (29%)	82 (73%)	94 (36%)	9 (0%)
Chloramphenicol	105 (70%)	106 (51%)	50 (31%)			72 (51%)	41 (34%)
Ceftriaxone	134 (14%)	131 (15%)	53 (40%)	39 (3%)			
Meropenem	17 (100%)	23 (70%)	16 (93%)	31 (19%)	18 (39%)		
Imipenem	130 (90%)	129 (82%)	51 (48%)	102 (4%)	76 (59%)		
Trimethoprim/Sulfamethoxazole	13 (0%)	16 (25%)	5 (17%)	4 (25%)		28 (4%)	

Gentamicin	135 (44%)	121 (41%)	62 (48%)	103 (24%)	78 (56%)	66 (76%)	
Erythromycin						66 (33%)	
Vancomycin							49 (14%)

Table S3. AMR variation by gender.

Antibiotics	Gender	Susceptibility	OR[95% CI]	*p*-Values
Ampicillin	Male	989 (15%)	1.23[0.921–1.637]	0.161
	Female	661 (13%)	Ref	
Amoxicillin/ Clavulanic acid	Male	723 (49%)	0.97[0.77–1.23]	0.82
	Female	456 (50%)	Ref	
Ciprofloxacin	Male	1599 (48%)	0.95[0.816–1.099]	0.475
	Female	1216 (49%)	Ref	
Chloramphenicol	Male	1073 (60%)	0.9[0.747–1.093]	0.295
	Female	776 (63%)	Ref	
Ceftriaxone	Male	593 (37%)	1.09[0.838–1.411]	0.528
	Female	424 (35%)	Ref	
Meropenem	Male	259 (79%)	0.72[0.447–1.16]	0.177
	Female	203 (84%)	Ref	
Imipenem	Male	1066 (86%)	1.1[0.839–1.435]	0.498
	Female	735 (85%)	Ref	
Trimethoprim/ Sulfamethoxazole	Male	1133 (22%)	0.84[0.678–1.034]	0.100
	Female	806 (26%)	Ref	
Gentamicin	Male	1340 (58%)	0.89[0.756–1.051]	0.172
	Female	1053 (61%)	Ref	
Erythromycin	Male	785 (30%)	0.75[0.595–0.941]	0.013
	Female	569 (36%)	Ref	
Vancomycin	Male	284 (40%)	1.01[0.687–1.487]	0.957
	Female	173 (40%)	Ref	
Oxacillin/ Methicillin	Male	137 (47%)	1.22[0.74–2.019]	0.432
	Female	113 (42%)	Ref	
